# Suicidal Behaviors Among United States Adolescents: Increasing Clinical and Public Health Challenges

**DOI:** 10.3390/children12010057

**Published:** 2025-01-03

**Authors:** Jhon Ostanin, Helena Miranda, Simon Shugar, Dina Abdo, Maria Carmenza Mejia, Charles H. Hennekens, Panagiota Kitsantas

**Affiliations:** 1 Herbert Wertheim College of Medicine, Florida International University, Miami, FL 33199, USA; josta002@fiu.edu; 2 Charles E. Schmidt College of Medicine, Florida Atlantic University, Boca Raton, FL 33431, USA; hdeazeredomi2023@health.fau.edu (H.M.); sshugar2018@health.fau.edu (S.S.);; 3 Department of Health Studies, University of Richmond, Richmond, VA 23173, USA; dina.abdo@richmond.edu; 4 Department of Population Health and Social Medicine, Charles E. Schmidt College of Medicine, Florida Atlantic University, Boca Raton, FL 33431, USA

**Keywords:** suicidal behaviors, adolescent psychiatry, mental health, high school students, United States

## Abstract

Background/Objectives: Suicide in the United States (US) adolescents is a major clinical and public health problem. In this original investigation, we explored trends in suicidal behaviors (ideation, planning, and attempts) among US adolescents from 2011 to 2021. Methods: The study sample included 90,306 adolescents from the 2011–2021 Youth Risk Behavior Surveillance System. Descriptive statistics and the chi-squared test were used to assess differences in suicidal behaviors across gender, race/ethnicity, and grade between 2011 and 2021. Results: The overall percentage of female adolescents reporting suicidal behaviors increased significantly between 2011 and 2021, and it was higher than males. In 2021, females exhibited significantly higher rates of considering suicide (30.0% vs. 14.3%), planning (23.6% vs. 11.6%), and attempts (13.3% vs. 6.6%) compared to their male counterparts. Whites were more likely to report suicidal ideation (22.7%) while Black non-Hispanic youth had a higher likelihood of making a suicide plan (17.7%), attempting suicide (14.5%), or making a suicide attempt requiring medical treatment (4.4%) relative to other racial/ethnic groups. Overall, ninth graders were more likely to report suicide attempts (11.6%) compared to 12th graders (8.6%). Conclusions: The results demonstrate significant increases in suicidal behavior among US adolescents, particularly in females. They suggest the need for gender-sensitive approaches in mental health support and prevention strategies. Overall, given the significant increase in suicidal behaviors, healthcare providers as well as public health professionals should prioritize mental health initiatives, promote awareness, and ensure access to mental health resources for adolescents.

## 1. Introduction

Suicide remains a significant public health challenge in the United States (US) and globally [1]. According to reports from the Centers for Disease Control and Prevention (CDC), there were 239,493 deaths between 2018 and 2022 in the US due to suicide, with a fatal injury rate of 13.9 per 100,000 people [2]. In 2021 alone, 48,183 suicides made it the 11th leading cause of death across all age groups [3]. Adolescents aged 10–19 are particularly vulnerable, with 2941 suicides recorded in 2021, ranking it as the third leading cause of death in this age group, only behind unintentional injury and homicide [2]. Additionally, suicidal behaviors in this demographic resulted in 117,314 hospitalizations, placing a significant burden on the healthcare system with over USD 19 billion in associated costs for nonfatal hospitalizations and emergency department visits [2].

Suicidal ideation, planning, and attempts represent critical warning signs that can precede fatal suicide events. Understanding these components and their demographic disparities is crucial to effective prevention efforts. Adolescents face unique vulnerabilities related to mental health. Research has shown that social and academic stressors, exposure to social media, and family-related pressures disproportionately affect young people, particularly females [4]. Males, while less likely to report suicidal ideation, often use more lethal methods, leading to a higher likelihood of fatal suicide attempts [5]. Additionally, disparities exist across racial and ethnic groups. For example, Black and Hispanic adolescents encounter systemic barriers to accessing culturally appropriate mental health resources, contributing to heightened risks of suicide attempts [6,7]. Protective factors, such as parental involvement, peer support, and access to school-based mental health services, have been shown to reduce these risks [8]. Understanding these intersecting factors is critical to designing effective prevention strategies.

Given the grave societal and health implications associated with suicidal behaviors and the increasing trends in younger populations, understanding the patterns of these behaviors in youth is crucial. The present study examined differences in suicidal behaviors between males and females as these groups often exhibit distinct patterns using data from the 2011–2021 Youth Risk Behavior Surveillance System. In addition, focused analyses of the most recent available data from 2021 were conducted to gain a deeper understanding of these behaviors within this vulnerable population.

## 2. Materials and Methods

### 2.1. Data Source and Analytic Sample

This study used data from the 2011–2021 YRBSS, a cross-sectional biannual, nationally representative survey of high school students in grades 9–12, conducted by the CDC since 1991. The YRBSS collects information from adolescents in both public and private schools across all 50 states and the District of Columbia. The YRBSS includes questions related to suicidal behaviors [9,10]. More details on YRBSS data collection methods can be found elsewhere [10].

The analytic sample for this study comprised data from six separate survey years, with 90,306 respondents; 2011 (*n* = 15,425), 2013 (*n* = 13,583), 2015 (*n* = 15,624), 2017 (*n* = 14,765), 2019 (*n* = 13,677), and 2021 (*n* = 17,232).

### 2.2. Measures

This study examined four variables related to suicidal behaviors: (1) seriously considered attempting suicide, (2) made a suicide plan, (3) attempted suicide, and (4) made a suicide attempt requiring medical treatment. Participants were asked the following questions in collecting data related to these measures: “During the past 12 months, did you ever seriously consider attempting suicide?”, “During the past 12 months, did you make a plan about how you would attempt suicide?”, “During the past 12 months, how many times did you actually attempt suicide?”, and “If you attempted suicide during the past 12 months, did any attempt result in an injury, poisoning, or overdose that had to be treated by a doctor or nurse?”. The first two questions (considered suicide and made a suicide plan) had “yes” or “no” response options. The third question (suicide attempts) was assessed by asking about the frequency of attempts: “0 times”, “1 time”, “2 or 3 times”, “4 or 5 times”, or “6 or more times”, but was later classified as “yes” or “no” for analytical purposes. The fourth question (making a suicide attempt requiring medical treatment) offered response options including “I did not attempt suicide during the past 12 months”, “yes”, or “no”. This variable was also classified as “yes” or “no”.

### 2.3. Data Analysis

The analysis of suicidal ideation and behavior involved examining associations between each of the four suicide behaviors and demographic characteristics such as gender (boy or girl), race/ethnicity (White non-Hispanic, Black non-Hispanic, Asian, Hispanic, or Multiple races), and grade (9, 10, 11, or 12). Chi-square tests with 95% confidence were performed to determine if there was a significant difference in each suicidal behavior across the selected demographic categories. Trends from 2011 to 2021 were examined comparing percentages of males and females for each suicide behavior.

## 3. Results

### 3.1. Trends in Suicidal Behaviors Between Males and Females from 2011 to 2021

**Figure 1:**
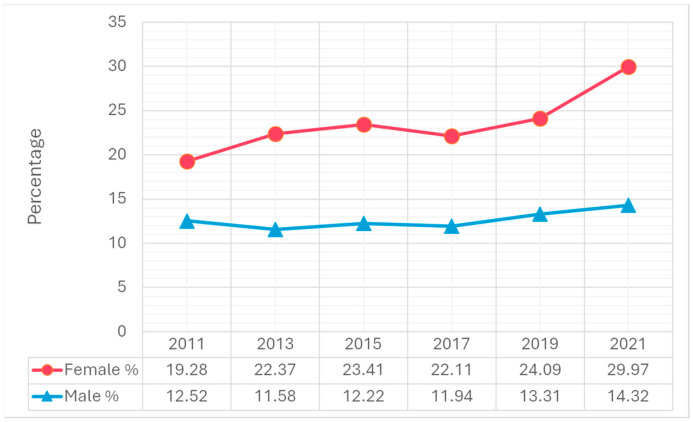
Differences in seriously considering suicide by gender, 2011–2021 Youth Risk Behavior Surveillance System.

**Figure 2:**
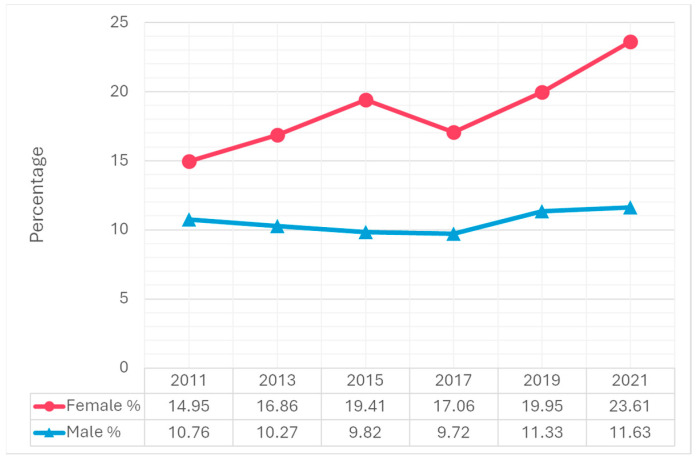
Differences in making a suicide plan by gender, 2011–2021 Youth Risk Behavior Surveillance System.

**Figure 3:**
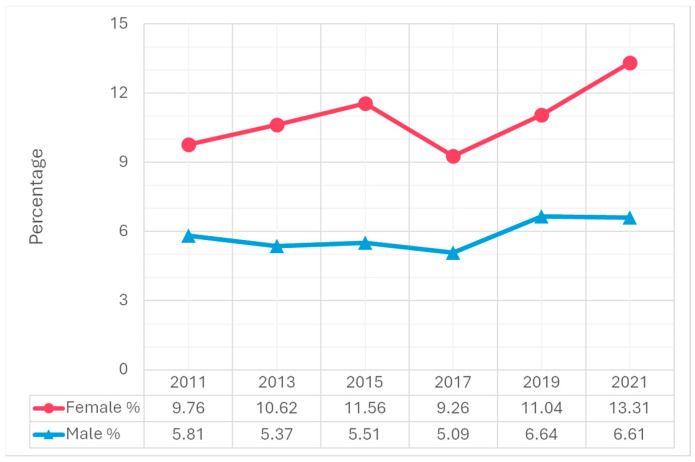
Differences in making a suicide attempt by gender, 2011–2021 Youth Risk Behavior Surveillance System.

**Figure 4:**
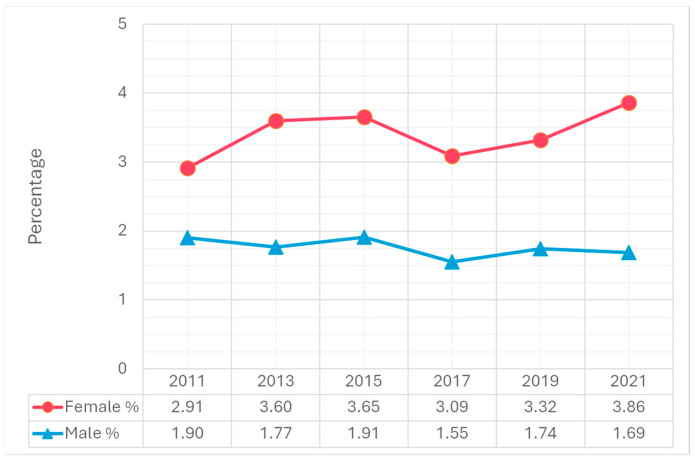
Differences in having an injurious suicide attempt by gender, 2011–2021 Youth Risk Behavior Surveillance System.

Trend analysis from 2011 to 2021 revealed significant increases in suicidal ideation, particularly among females. The percentage of females who seriously considered suicide increased from 19.3% in 2011 to 30.0% in 2021, compared to a smaller increase among males, from 12.5% to 14.3% (Figure 1). A similar trend was observed in suicide planning. The percentage of females who made suicide plans rose from 14.9% in 2011 to 23.6% in 2021. Among males, the percentage increased slightly from 10.8% in 2011 to 11.6% in 2021 (Figure 2). Attempting suicide among females increased 1.4-fold from 2011 to 2021. Among males, the percentage increased slightly from 5.8% in 2011 to 6.6% in 2021 (Figure 3). The percentage of females who had an injurious suicide attempt also exhibited an overall upward trend from 2.9% in 2011 to 3.9% in 2021, whereas the percentage of males slightly decreased from 1.9% in 2011 to 1.7% in 2021 (Figure 4).

### 3.2. Suicidal Behaviors in Males and Females in 2021

In 2021, 22.2% of adolescents considered seriously attempting suicide, 17.6% made a suicide plan, 10.2% reported attempting suicide, and 2.9% made a suicide attempt that required medical treatment (Table 1). Females were significantly more likely to report these behaviors than males. For example, females (30.0%) were more than twice as likely as males (14.3%) to seriously consider attempting suicide (*p* < 0.001). In addition, during the same year, 23.6% of females made a suicide plan, which was significantly higher than males (11.6%) (*p* < 0.001).

### 3.3. Suicidal Behaviors by Race/Ethnicity and Grade Across Males and Females in 2021

White females (31.4%) and males of multiple races (18.1%) were more likely to seriously consider attempting suicide (Table 2). In addition, making a suicide plan was more prevalent among Hispanic females (24.8%) and males of multiple races (20.5%). Black adolescents, however, were more likely to attempt suicide and make a suicide attempt that required medical treatment compared to other racial groups.

A significantly higher percentage of females in 10th grade (33.6%) and males in 11th (16.8%) and 12th (16.5%) grades reported seriously considering attempting suicide. Making a suicide plan was more prevalent among 10th-grade females (26.9%) and 11th-grade males (13.7%). In addition, attempting suicide was particularly higher among 9th (15.8%) and 10th (15.6%) grade females, and making a suicide attempt that required medical treatment was also higher in these grades (4.8% for 9th grade and 4.1% for 10th graders).

### 3.4. Suicidal Behaviors by Race/Ethnicity for the Entire Sample in 2021

Suicidal behaviors also varied across racial/ethnic groups regardless of gender. Seriously considering suicide was particularly high among youth of multiple races (24.1%), followed by White, non-Hispanic (22.7%), Hispanic (22.0%), and Black non-Hispanic (21.6%). Asian youth had the lowest percentage at 17.7 (Table 2).

Adolescents of multiple races (24.4%) and Hispanic origin (18.7) were significantly more likely to report making a suicide plan (*p*-value < 0.05). However, attempting suicide and making a suicide attempt that required medical treatment was significantly higher among Black adolescents (14.5% and 4.4%, respectively).

### 3.5. Suicidal Behaviors by Grade for the Entire Sample in 2021

A significantly higher percentage of 10th and 11th graders reported seriously considering attempting suicide (around 23.0%, *p*-value = 0.013) and made a suicide plan (around 18.0%, *p*-value = 0.006) (Table 2). Attempting suicide was also significantly higher in 9th (11.6%) and 10th (10.9%) graders compared to the other grades (*p*-value < 0.001).

## 4. Discussion

This study underscores significant disparities in suicidal ideation and behaviors among US adolescents, particularly by gender, race/ethnicity, and grade level. Findings indicate that females consistently exhibit higher rates of suicidal ideation, planning, and attempts than their male counterparts, a trend that aligns with previous studies showing females are more likely to internalize distress, often resulting in suicidal ideation and behaviors [1,10]. However, males, while less likely to report ideation, often use more lethal methods, leading to a higher likelihood of fatal suicide attempts [5]. This gender-based difference indicates the need for tailored prevention strategies that address the unique risks faced by each group.

The significant increase in suicidal ideation among females, from 19.3% in 2011 to 30.0% in 2021, suggests that factors such as social pressures, exposure to social media, and pandemic-induced isolation may exacerbate mental health vulnerabilities. Research shows that adolescent females may experience social and academic stressors differently, particularly during adolescence when social comparisons and pressures to conform are heightened [4,11]. Moreover, the COVID-19 pandemic has contributed to a decline in mental health, disproportionately affecting females due to greater social isolation, disrupted support networks, and heightened family responsibilities [12,13].

Racial and ethnic disparities further compound the issue, with Black and Hispanic youth at greater risk. Black non-Hispanic adolescents reported the highest rates of suicide attempts and medically treated attempts, with females in this group showing especially high percentages. This aligns with national trends indicating a growing mental health crisis among Black youth, potentially exacerbated by socioeconomic stressors, systemic discrimination, and limited access to culturally responsive mental health resources [6,7]. Hispanic adolescents also exhibited elevated rates of suicidal ideation, planning, and attempts, suggesting unique vulnerabilities within this population that may include acculturative stress, language barriers, and stigma around mental health [14,15]. The high prevalence of suicidal ideation among White non-Hispanic adolescents, however, suggests that risk factors such as academic and social pressures may affect them differently [4]. These findings highlight the critical need for equitable mental health interventions, including culturally tailored prevention efforts that address the unique stressors and mental health barriers faced by each racial and ethnic group.

The age-related findings indicate that younger students, particularly ninth and tenth graders might be more vulnerable to suicidal behaviors compared to their older counterparts. This is consistent with existing literature suggesting that the transition into high school presents unique challenges, such as adaptation to new social environments, academic expectations, and potential exposure to bullying or cyberbullying [16]. The developmental immaturity of younger adolescents may further impede effective coping mechanisms, showing the importance of early school-based interventions to build resilience and coping skills among this vulnerable age group [8].

A notable study finding highlights a stark gender disparity in medically treated suicide attempts, with 3.9% of females requiring medical intervention compared to 1.7% of males. This disparity suggests that females are more likely to engage in nonfatal suicide attempts that nevertheless result in injuries serious enough to require professional intervention. The higher rates of medically treated attempts among females underline the importance of timely and accessible care, especially for youth lacking robust support systems outside school environments. Public health initiatives should prioritize timely and accessible medical and psychological care for females in particular, as they may not always have access to support systems outside of school settings [12,17]. School-based health services could play a vital role in providing immediate mental health support, particularly for adolescents with limited access to external resources. Expanding these services and training school personnel to recognize early warning signs could significantly reduce risks.

While this study offers valuable insights into the trends and disparities in adolescent suicidal behaviors, several limitations must be acknowledged. The YRBSS data are self-reported, which introduces the possibility of response bias, as students may underreport or exaggerate their behaviors due to social stigma or fear of reprisal. Furthermore, the cross-sectional nature of the survey prevents the identification of causal relationships or long-term trends in suicidal behaviors. Future research should employ longitudinal designs to better understand the causal pathways that contribute to suicidal behaviors over time and examine the interplay between risk and protective factors across demographic subgroups. [10,18].

The findings highlight the urgency of implementing targeted mental health interventions tailored to adolescents’ specific needs. Given the disparities observed, interventions should incorporate culturally and gender-sensitive interventions that prioritize the needs of underserved populations, particularly Black, Hispanic, and female youth. Integrating mental health education into school curricula, promoting awareness of mental health resources, and providing training for school personnel to recognize and respond to signs of suicidal behavior could significantly reduce the risk of suicide among adolescents [17].

Future research should explore longitudinal methods to capture long-term trends in adolescent mental health and examine causal relationships. Additionally, studies examining the impact of social media and digital interactions on adolescent mental health, particularly among females, could provide valuable insights for developing effective preventive strategies in an increasingly digital age [19].

## 5. Conclusions

This study reveals a troubling landscape of adolescent mental health disparities in the US, with female, Black, Hispanic, and younger students particularly vulnerable to suicidal behaviors. These findings reveal the need for urgent, targeted interventions that prioritize accessibility and cultural responsiveness. Addressing adolescent mental health disparities requires a multifaceted approach, involving schools, families, communities, and public health systems. Collaboration among these stakeholders is essential to creating sustainable solutions that incorporate culturally relevant prevention and intervention efforts. By implementing tailored, culturally sensitive, and school-based mental health programs, we can work toward reducing adolescent suicide rates and fostering resilience among youth. These programs should include comprehensive training for school personnel, integration of mental health curricula, and the expansion of school-based health services to ensure early detection and support. Investing in these initiatives is essential to mitigating the growing mental health crisis and building a supportive, mentally healthy environment for all adolescents. Policymakers and healthcare professionals must act decisively to allocate resources and develop frameworks that promote mental health equity and resilience among vulnerable youth.

## Figures and Tables

**Table 1:** Suicidal Behaviors and Differences between Female and Male Adolescents, 2021 Youth Risk Behavior Surveillance System.

Suicidal Behavior	Total % (95% CI) *n* = 17,232	Female % (95% CI) *n* = 8289	Male % (95% CI) *n* = 8943	*p*-Value
**Seriously considered attempting suicide**				<0.001
Yes	22.2 (21.1–23.3)	30.0 (28.5–31.4)	14.3 (13.3–15.4)	
No	77.8 (76.7–78.9)	70.0 (68.6–71.5)	85.7 (84.6–86.7)	
**Made a suicide plan**				<0.001
Yes	17.6 (16.4–19.0)	23.6 (22.1–25.1)	11.6 (10.5–12.8)	
No	82.4 (81.0–83.6)	76.4 (74.9–77.9)	88.4 (87.2–89.5)	
**Attempted suicide**				<0.001
Yes	10.2 (9.4–11.0)	13.3 (12.0–14.7)	6.6 (5.8–7.5)	
No	89.8 (89.0–90.6)	86.7 (85.3–88.0)	93.4 (92.5–94.2)	
**Made a suicide attempt requiring medical treatment**				<0.001
Yes	2.9 (2.5–3.4)	3.9 (3.1–4.8)	1.7 (1.4–2.0)	
No	97.1 (96.6–97.5)	96.1 (95.2–96.9)	98.3 (98.0–98.6)	

**Table 2:** Differences in Suicidal Behaviors by Race/Ethnicity, Grade and Across Female and Male Adolescents, 2021 Youth Risk Behavior Surveillance System.

Behavior	Female % (95% CI)	*p*-Value	Male % (95% CI)	*p*-Value	Total % (95% CI)	*p*-Value
**Seriously considered attempting suicide ** *n* = 16,927						
**Race/Ethnicity**		0.014		0.063		0.007
White, non-Hispanic	31.4 (29.2–33.7)		14.5 (13.1–16.0)		22.7 (20.9–24.6)	
Black, non-Hispanic	30.5 (25.6–36.0)		13.0 (10.4–16.0)		21.6 (18.4–25.2)	
Asian	24.2 (19.8–29.1)		11.8 (8.9–15.4)		17.7 (15.5–20.2)	
Hispanic	28.7 (26.8–30.8)		14.2 (12.1–16.6)		22.0 (20.8–23.3)	
Multiple races	28.5 (22.7–35.2)		18.1 (14.5–22.4)		24.1 (20.0–28.7)	
**Grade**		<0.001		<0.001		0.013
9	30.7 (27.4–34.3)		11.9 (9.8–14.5)		21.2 (18.9–23.6)	
10	33.6 (30.7–36.7)		12.7 (11.2–14.4)		23.2 (21.1–25.3)	
11	29.7 (26.8–32.8)		16.8 (15.0–18.7)		23.3 (21.6–25.1)	
12	25.6 (23.8–27.4)		16.5 (14.2–19.0)		21.1 (19.6–22.6)	
**Made a suicide plan ** *n* = 16,321						
**Race/Ethnicity**		0.579		0.162		0.020
White, non-Hispanic	22.9 (20.7–25.3)		11.2 (9.8–12.8)		16.9 (15.2–18.8)	
Black, non-Hispanic	24.3 (20.7–28.2)		11.3 (8.7–14.5)		17.7 (15.0–20.8)	
Asian	22.9 (19.5–26.6)		10.3 (6.5–15.8)		16.5 (13.4–20.3)	
Hispanic	24.8 (22.6–27.0)		11.9 (9.9–14.3)		18.7 (17.1–20.5)	
Multiple races	15.0 (10.9–20.4)		20.5 (16.5–25.1)		24.4 (19.3–30.5)	
**Grade**		<0.001		<0.001		0.006
9	25.1 (22.3–28.3)		11.4 (9.3–14.0)		18.2 (16.1–20.5)	
10	26.9 (24.8–29.1)		9.8 (8.0–12.0)		18.4 (16.6–20.3)	
11	22.4 (19.2–26.0)		13.7 (12.1–15.5)		18.0 (16.3–19.9)	
12	19.6 (17.9–21.4)		11.7 (9.9–13.8)		15.7 (14.2–17.3)	
**Attempted suicide ** *n* = 15,573						
**Race/Ethnicity**		<0.001		<0.001		<0.001
White, non-Hispanic	12.4 (10.7–14.5)		5.5 (4.5–6.7)		9.0 (7.8–10.5)	
Black, non-Hispanic	17.8 (14.1–22.3)		11.2 (8.4–14.7)		14.5 (11.9–17.5)	
Asian	8.3 (4.9–13.8)		4.7 (2.3–9.4)		6.4 (4.3–9.4)	
Hispanic	13.8 (12.0–15.9)		6.5 (4.3–9.8)		10.7 (9.5–12.0)	
Multiple races	13.9 (10.6–18.0)		8.1 (5.4–12.0)		11.7 (9.1–14.9)	
**Grade**		<0.001		0.657		<0.001
9	15.8 (13.8–18.0)		6.9 (5.4–8.8)		11.6 (10.3–13.0)	
10	15.6 (13.3–18.2)		6.2 (5.0–7.6)		10.9 (9.5–12.4)	
11	11.2 (8.8–14.1)		6.2 (4.6–8.3)		8.9 (7.5–10.5)	
12	10.3 (8.7–12.1)		6.9 (5.8–8.0)		8.6 (7.7–9.6)	
**Made a suicide attempt requiring medical treatment ** *n* = 12,083						
**Race/Ethnicity**		0.027		<0.001		<0.001
White, non-Hispanic	3.5 (2.8–4.5)		1.2 (0.9–1.8)		2.4 (1.9–3.1)	
Black, non-Hispanic	5.5 (3.5–8.7)		3.3 (1.9–5.6)		4.4 (3.1–6.3)	
Asian	2.2 (0.9–5.2)		1.1 (0.3–3.4)		1.6 (0.6–4.1)	
Hispanic	4.7 (3.6–6.0)		2.0 (1.5–2.7)		3.8 (3.3–4.5)	
Multiple races	3.0 (1.5–5.9)		3.1 (1.3–7.0)		3.0 (1.8–4.9)	
**Grade**		0.089		0.062		0.388
9	4.8 (3.8–6.0)		1.1 (0.6–2.4)		3.1 (2.5–3.8)	
10	4.1 (2.9–5.8)		1.8 (0.9–3.4)		2.9 (1.9–4.3)	
11	3.3 (2.4–4.6)		2.3 (1.4–3.7)		3.0 (2.3–3.9)	
12	3.2 (2.1–4.8)		1.5 (0.9–2.3)		2.4 (1.9–3.1)	

## Data Availability

The data presented in the study are openly available in the Youth Risk Behavior Survey at https://nccd.cdc.gov/Youthonline/App/Default.aspx (accessed on 3 June 2024).

## References

[1] (2021). Suicide Worldwide in 2019 Global Health Estimates.

[2] CDC WISQARS—Web-Based Injury Statistics Query and Reporting System. https://wisqars.cdc.gov/.

[3] Provisional Mortality Statistics, 2018 Through Last Week Request. https://wonder.cdc.gov/mcd-icd10-provisional.html.

[4] Twenge J.M., Cooper A.B., Joiner T.E., Duffy M.E., Binau S.G. (2019). Age, period, and cohort trends in mood disorder indicators and suicide-related outcomes in a Nationally Representative Dataset, 2005–2017. J. Abnorm. Psychol..

[5] Shain B., Braverman P.K., Adelman W.P., Alderman E.M., Breuner C.C., Levine D.A., Marcell A.V., O’Brien R.F. (2016). Suicide and suicide attempts in adolescents. Pediatrics.

[6] Bridge J.A., Horowitz L.M., Fontanella C.A., Sheftall A.H., Greenhouse J., Kelleher K.J., Campo J.V. (2018). Age-related racial disparity in suicide rates among US youths from 2001 through 2015. JAMA Pediatr..

[7] Hoffmann J.A., Alegría M., Alvarez K., Anosike A., Shah P.P., Simon K.M., Lee L.K. (2022). Disparities in pediatric mental and behavioral health conditions. Pediatrics.

[8] Hawton K., Saunders K.E., O’Connor R.C. (2012). Self-harm and suicide in adolescents. Lancet.

[9] 2021 National Youth Risk Behavior Survey. https://www.cdc.gov/yrbs/dstr/pdf/YRBS_Data-Summary-Trends_Report2023_508.pdf.

[10] Mpofu J.J., Underwood J.M., Thornton J.E., Brener N.D., Rico A., Kilmer G., Harris W.A., Leon-Nguyen M., Chyen D., Lim C. (2023). Overview and methods for the youth risk behavior surveillance system—United States, 2021. MMWR Suppl..

[11] Keyes K.M., Gary D., O’Malley P.M., Hamilton A., Schulenberg J. (2019). Recent increases in depressive symptoms among US adolescents: Trends from 1991 to 2018. Soc. Psychiatry Psychiatr. Epidemiol..

[12] Moutier C. (2020). Suicide Prevention in the COVID-19 Era: Transforming Threat Into Opportunity. JAMA Psychiatry.

[13] Zalsman G., Stanley B., Szanto K., Clarke D.E., Carli V., Mehlum L. (2020). Suicide in the time of COVID-19: Review and recommendations. Arch. Suicide Res..

[14] Concepcion Zayas M.T., Fortuna L.R., Cullins L.M. (2019). Depression in Latino and immigrant refugee youth. Child Adolesc. Psychiatr. Clin. N. Am..

[15] Ramirez A.G., Gallion K.J., Aguilar R., Dembeck E.S. (2017). Mental Health and Latino Kids: A Research Review. https://salud-america.org/wp-content/uploads/2017/09/FINAL-mental-health-research-review-9-12-17.pdf.

[16] Espelage D.L., Holt M.K. (2013). Suicidal ideation and school bullying experiences after controlling for depression and delinquency. J. Adolesc. Health.

[17] Ajluni V., Amarasinghe D. (2024). Youth suicide crisis: Identifying at-risk individuals and prevention strategies. Child Adolesc. Psychiatry Ment. Health.

[18] Turecki G., Brent D.A. (2016). Suicide and suicidal behaviour. Lancet.

[19] Nesi J. (2020). The impact of social media on Youth Mental Health. North Carol. Med. J..

